# The Thirty-First Long Fox Memorial Lecture: The Experimental Study of Development

**Published:** 1942

**Authors:** C. M. Yonge

**Affiliations:** Professor of Zoology in the University of Bristol


### The Bristol Medico-Chirurgical Journal

*" Scire est nescire, nisi id me*
*Scire alius sciret."*

AUTUMN, 1942.

### THE THIRTY-FIRST LONG FOX MEMORIAL LECTURE:

BY

PROFESSOR C. M. YONGE, D.Sc.,
*Professor of Zoology in the University of Bristol.*

DELIVERED IN THE UNIVERSITY OF BRISTOL ON TUESDAY, 7th JULY, 1942,

ON

### THE EXPERIMENTAL STUDY OF DEVELOPMENT.

THE problem of development—the manner in which an organism attains the size and elaboration of its adult structure—was not unnaturally a matter which early excited the interest of man. There exist, indeed, few more fascinating subjects than the change, over a period of hours, days, weeks, at most a few months, from the apparently simple structure of the fertilized egg to the immense structural and functional complexity of the insect, fish, bird or man into which it develops. Yet so commonplace is the fact of development that we tend to take for granted a process occupying days or weeks, while the occurrence of evolutionary processes over hundreds of millions of years remains a subject of dispute.

The foundations of the science of embryology, that branch of biology which deals with development, were laid by the Greeks. Amongst the Hippocratic writings there are many allusions to development, although mainly in connexion with obstetrical and gynæcological problems. But even at this early date, in the fourth century B.C., there are attempts at causal explanations and the important fact was noted that the embryo " dries up," *i.e.* loses water during development. But it is from Aristotle, the first great biologist, in some respects the greatest of all biologists, that the bulk of classical knowledge on development comes. His work on *The Generation of Animals* is the first text-book on embryology, beyond which little further advance was made until modern times. Yet, in the absence of the essential aids of microscope and microtome, the more fundamental facts inevitably escaped him. Thus, although he distinguished between oviparity, ovo-viviparity and viviparity, described later stages in development and allotted the correct functions to the placenta and the umbilical cord in the mammals, he knew nothing of the essential nature of the egg and the spermatozoon. Adopting what appears to have been an Egyptian view, he regarded the human embryo as arising from the menstrual blood under the moulding and dynamic agency of the male secretions, although he admitted the possibility of the operation of some previously existing mechanism which was thus set in motion. In one important matter he anticipated modern views. The Hippocratic belief was that development was no more than a revelation of previously existing complexity, *i.e.* essentially merely an increase in size, a belief later known as preformation : but Aristotle concluded that development represents an ever-increasing degree of complexity of structure ; he was an upholder of the opposing view of epigenesis.

Galen, the great physician of the Roman world, made comparatively minor contributions to embryology and we have to await the revival of the spirit of scientific enquiry at the Renaissance for further significant advance. Leonardo da Vinci, one of the supreme minds of all times, left in his amazing note-books beautiful drawings of the human fœtus in utero, but, more significant of the new age of which he was the precursor, he made measurements of embryos at various stages and, by his mathematical analysis of the processes of development and growth, has claims to being considered the first experimental embryologist. William Harvey, whose fame as an embryologist has been overshadowed by his still greater fame as discoverer of the circulation of the blood, maintained in his great work on *The Generation of Animals* that "all animals are in some sort produced from eggs" wherein "no part of the future organism exists *de facto*, but all parts lie here *in potentia*."

Here was a clear statement of embryological fact and from such a beginning, with the help of the recently-discovered microscope, rapid progress might have been expected. But, most unfortunately, a controversy developed which impeded progress for more than a century. Malpighi, the first great histologist, made microscopical examination of the developing egg of the hen, but did so in the heat of the Italian summer when development may proceed without incubation. Hence he found, in what he took to be the undeveloped egg, clear traces of the future adult and so claimed to have demonstrated the fact of preformation which Harvey had denied. About the same time the Dutch microscopist, Leeuwenhoek, identified spermatozoa from the seminal fluid and, seeing faint indications of internal structure, claimed that they were miniatures of the adult. Thus there arose two schools amongst the preformationists; the ovists who proclaimed that the adult structure was present in the egg and the animalculists who claimed that it resided within the sperm. And until the end of the eighteenth century the history of embryology is largely the record of the wordy battles between the upholders of these views and between them and the supporters of epigenesis. Such progress as was made concerned such matters as the identification of the ovaries and the testes.

The light of scientific truth re-emerges in 1759 with the publication by Caspar Friedrich Wolff of his *Theoria Generationis*, in which he stated that the parts of the embryo of the chick appeared in turn and could be *seen being formed*. His work leads directly to that of the greatest of embryologists, the Esthonian, Karl Ernst von Baer, whose work in descriptive and comparative embryology brings us almost to modern times; he died at a great age in 1876. Meanwhile Spallazani, the pioneer experimental zoologist of the eighteenth century, had shown that sperms were essential to development. But it was not until the middle of the last century that the union of the egg and the sperm was observed and still later before it was realized that this involved the union of the nuclei. From this discovery, in turn, there dates the beginning of those investigations which have culminated in the discovery, first of the chromosomes within the nucleus, and then of the genes borne upon these which, linking up with Mendel's experimental study of inheritance, have been revealed as the controlling agencies in heredity.

To revert to the descriptive work of von Baer and his followers in the nineteenth century. It was shown that the developmental history of any animal was an orderly series of stages, beginning with the fertilized egg which divided repeatedly to form typically a hollow sphere known as the blastula, which by intucking of the cells on one side, or overgrowth by the future outer cells, if the former process was mechanically impossible, produced a two-layered stage, the gastrula. The outer layer of cells became known as the ectoderm, and gave rise to the skin, nervous system and sense organs; the inner, which formed the future gut, as the endoderm. With the formation of the gastrula the first indications of the future organs—in the vertebrates the nervous system and the primitive axial skeleton or notochord—made their appearance. Between the two primary cell layers was introduced an intermediate mesoderm layer from which were derived in development the body cavity, the remainder of the skeleton, and the muscular, circulatory, excretory and reproductive systems. After this initial blocking out in the rough of the future organs there followed a period of tissue differentiation, at the conclusion of which the developing organism became a functional individual.

The development of great numbers of animals, both vertebrates and invertebrates, was studied in detail during the latter half of the last century and a most imposing mass of knowledge acquired, much of it in connection with the study of evolutionary processes initiated by Darwin who had published his *Origin of Species* in 1859.

But in any science description is only the first stage ; it is the prelude to analysis by measurement and experiment. The distinguished American embryologist, Professor F. G. Conklin, who played a notable part in the initiation of the experimental period in embryology summarized development as a series of responses to stimuli, the responses being not merely repetitive as in the case of the tissue cells. He likened the egg to a complex machine so constructed that it transforms itself at every critical stage into another machine with its own peculiar mode of action. The problems to be solved concerned the nature of the mechanisms within this continually changing machine.

Fertilization of the egg became recognized as a dual process, one of activation, *i.e.* the initiation of cleavage, and the other consisting of the union of the male and female nuclei, from the sperm and egg respectively. The latter process ensured the passage into the new organism, so initiated, of hereditary material from two sources, but, although in the majority of cases entrance of the sperm is also necessary for activation, important exceptions reveal that the egg can develop without the presence of the sperm. In representatives of various groups of animals, notably in the Insecta and Crustacea, eggs frequently develop parthenogenetically, *i.e.* without the assistance of the males which may be absent during certain periods of the life-history (in a few cases no males have been found at any stage). Normally, however, a series of parthenogenetic generations is followed by one in which males appear. In other groups, notably the Echinodermata, artificial activation is possible. Jacques Loeb, one of the greatest of Jewish emigrants from Germany to the United States, showed how the eggs of starfish can be stimulated to develop by treatment, causing first a temporary increase in the permeability of the egg, followed by the removal of some of the water from within it. As a result of activation the potentialities of the quiescent egg cell are dramatically converted into kinetic energy, revealed in some cases by an immediate great increase in oxygen consumption, but invariably by the subsequent initiation of the process of cleavage.

A problem which early presented itself in this period of experimental analysis was the fate of the individual cells into which the single activated egg cell gave rise in early cleavage. Did cleavage, it was asked, divide the egg into portions corresponding to the parts of the future embyro, *i.e.* were definite organ-forming substances present in different parts of the egg and so became separated from one another when the egg divided ? Experiments were carried out in which the early cleavage products, or blastomes, were separated from one another and their subsequent fate followed. These revealed that eggs could be divided into two types. There were *mosaic* eggs in which any portion of the products of early cleavage formed only that portion of the embyro which it would have formed had it remained in contact with the other product or products of egg division. But there were other, so-called *regulative*, eggs in which separated blastomeres formed complete but small embryos. Partial separation of the blastomeres produced double monsters ; the union of two activated eggs led to the formation of an unusally large, but single, embyro. Thus in eggs of this type, which included those of the Echinodermata and, to a more limited extent, of the Amphibia, the early stages of development are plastic ; the greater part, though not actually all, of the egg has no predestined fate. But invariably this plastic stage is succeeded by a mosaic stage in which the fate of the various parts is determined. This comes, in the case of the Amphibia, at about mid-gastrulation. And it is upon amphibian development that much of the significant work of the past twenty years has been done.

The amphibian egg is not homogeneous. It is rich in yolk, but the greater part of this collects at one end, the so-called " vegetative " pole, the bulk of the protoplasm with the nucleus lying near the opposite " animal " pole. It was discovered at a relatively early stage in embryological investigation that, although before fertilization the egg is apparently radially symmetrical about an axis which passes from the animal to the vegetative pole, bilateral symmetry appears immediately after fertilization. The animal pole is dark in colour, the vegetative pole light, but between the two after the entrance of the sperm there extends a grey crescent. The position of this has been regarded as controlled by the position of entrance of the sperm because it usually appears exactly opposite to this ; but there is some doubt on this point. What is certain, however, is that from this stage onwards the orientation of the developing egg and so of the future embyro and adult is determined. The widest area of the grey crescent is mid-dorsal and the egg is now bilaterally symmetrical about a plane passing through the middle of the crescent and the two poles. The first division of the egg is normally in this plane. The result of later cleavage is to bring about the formation of a hollow blastula composed of many small cells on one side, derivatives of the micromeres from the animal pole and, on the other, of larger cells from the yolk-laden macromeres of the vegetable pole.

The fate of the cells of the blastula has been followed out by the ingenious method of staining them in life with harmless dyes such as neutral red or Nile blue. In this way the superficial cells at this stage in the developing amphibian—frog or newt—have been mapped out into "presumptive" areas. The term presumptive is important. It implies that, under normal developmental conditions, these areas will develop into certain specified structures, *e.g.* endoderm, mesoderm, notochord, neural plate, or epidermis. But actually most are still fundamentally undetermined and, as we shall see, under certain experimental conditions, or as a result of damage, their fate may be changed. Of one region only is the destiny irrevocably determined at this stage and that is the area occupied by the cells of the grey crescent.

The extraordinary significance of this region was discovered by the late Professor H. Spemann, who was subsequently awarded the Nobel Prize for his work. He developed a very beautiful technique whereby he was able to excise portions of these early embryos and graft them into other embryos. Carefully executed experiments and critical analysis of the results obtained led him to one of the really significant advances in the experimental analysis of development.

Spemann used the embryos of newts, which provide better experimental material than those of frogs. When the gastrula is formed—by a combination of overgrowth and inpushing in the amphibia—the opening of the new internal cavity, lined by endoderm, constitutes the blastopore. In animals where little yolk is present in the egg this represents a rounded opening, but in the amphibia, where the egg is yolky, this is blocked by a plug of yolk-laden cells. The blastopore comes to lie on the posterior side of the embryo and from its upper, dorsal margin there extends forward the shallow medullary groove which later forms the neural tube. From this, by subsequent specialization of the cells, is formed the central nervous system characteristic of all vertebrates. Spemann removed the upper lip of the blastopore from one embryo and grafted this into the side of another embryo at the same stage in development and established the remarkable fact that in front of the grafted cells there appeared a medullary groove in addition to the one which appeared in the normal position. Meanwhile in the original embryo from which the dorsal lip had been removed no medullary groove appeared. He further showed that these results could only be obtained if the tissue was removed and the graft established in young gastrulæ. Later removal did not affect the subsequent appearance of the medullary groove and grafting had no effect. At the same time the experiment had not to be carried out too soon or no effect was produced by the grafted cells.

These results were explained by Spemann by postulating the presence of an "organizer" in the dorsal lip of the blastopore, the presence of which induced the formation of the medullary groove. An invisible, chemical change in the cells preceded the visible structural differentiation, so that if the dorsal lip was removed after a certain stage in development the medullary groove would still appear. In the same way the cells of the embryo into which the excised dorsal lip was grafted had to be "competent"; if the graft was made too late the fate of these cells, which would normally develop into skin, had been determined and the organizer could not therefore induce the formation of an additional medullary groove.

Later work, some of it in this country, notably by Needham and Waddington, has abundantly confirmed these results. An organizer or "organization centre" has been located in the embryos of other amphibia and other groups of vertebrates, such as mammals, birds and fishes, but also in the embryos of invertebrates such as echinoderms and insects. It was early shown that induction of the formation of a medullary groove would take place, not only after the cells had been narcotized but also that the organizing substance, or evocator, as it is now frequently termed, persists after the death of the cells containing it. It is resistant to heat, to freezing and to the action of a wide range of organic substances; indeed it is only destroyed when the cells are reduced to ash. That a chemical substance is involved is indicated by the fact that small pieces of agar after being placed in contact with the dorsal lip of the blastopore for a short time acquire the power of inducing a medullary groove when applied to other embryos, indicating that some specific substance had diffused into the agar from the cells of the original embryo. A variety of artificial evocators such as methylene blue, sterols and fatty acids have a similar effect, and it is probable that they produce their effect by causing the cells of the embryo to release the normal evocating substance. Attempts to isolate the chemical substance, or substances, involved have not, as yet, been completely successful. In view of the minute quantities concerned this is not surprising. But the evocator does appear to be soluble in ether and petrol-ether and it may be one of the sterols. If this is the case, then it belongs to the same group of substances as certain of the sex hormones and cancer-producing agents. All of these, it will be noted, are in some way or other concerned with development or growth—normal or pathological.

The organizer is not specific; cells from the primary organization centre of the embryo of one group of animals, for instance birds, will induce the formation of a medullary groove in the embryos of another, such as amphibia. But there is something inherent within the cells of the particular embryo which insures that it is amphibian tissues which are induced by the evocator and not avian. It may be assumed that it is the genes resident on the chromosomes which are responsible for the particular reactions of the cells—the stimulating substance being identical in all cases. There is certainly a most complex interplay between the stimulating evocator and the reacting cells, the analysis of which will involve much further work.

There is evidence that the pattern which the induced tissues assume is influenced both by the evocator and the tissues affected.

The position of the primary organization centre was determined, as we have seen, at fertilization. It invariably appears within the region of the grey crescent. The actual position of this would seem to be determined by what, in our present state of knowledge, we can only describe as the interaction of certain gradients within the egg. There is a gradient from the vegetative pole to the animal pole and apparently at right angles to this a second gradient, the organization centre appearing at the intersection of the two.

Although the medullary groove, the first indication of the future central nervous system, is the first organ rudiment to be *induced* it is actually not the first to be *formed.* Beneath it, along the upper side of the archenteric, or primitive gut, cavity which is formed at gastrulation by the intucking of the cells around the blastopore, two other organ rudiments make their appearance. These are the notochord, the primitive axial skeleton of the vertebrates, and the segmented mesodermal somites which give rise to the body cavity, body musculature, excretory system and so forth. The notochord is situated in the antero-posterior axis of the embryo, the mesodermal somites appearing bilaterally on either side of this. These structures arise spontaneously at a certain critical stage in development, namely mid-gastrulation, and it is their presence which induces the appearance of the medullary groove above them. The dorsal lip of the blastopore actually consists of cells which will, by the continual process of intucking, form notochord and mesoderm.

This induction can be proved in two ways. First by grafting early notochordal and mesodermal cells below "competent" ectoderm, following which a medullary plate invariably makes its appearance above the region of the graft. Second by rearing suitably susceptible embryos in dilute saline solutions. This treatment has the effect on converting the normal gastrulation invagination into an evagination, *i.e.* the cells which form the primitive gut, notochord and mesoderm are pushed out away from the rest of the embryo instead of being tucked in under the superficial ectoderm. Under these conditions a notochord and mesodermal plates lateral to this appear within the mass of cells which has been pushed out, but no medullary plate is formed along the mid-dorsal line of the ectoderm because there is no chorda-mesoderm below it and so no inductive action. We speak, therefore, of the *self-differentiation* of the chorda-mesoderm and of the *induction* of the medullary plate which normally appears above it.

The result of the appearance of the chorda-mesoderm and of the inductions which this primary organization centre brings about is thus the formation of a zone of constructive activity along the antero-posterior axis of the embryo. This has become known as the primary organ field. This conception of fields of dynamic activity is at present no more than a description of what we observe and is in no way an explanation, although there are many indications that some underlying physical mechanism will eventually be disclosed. The primary organ field gives rise to a series of secondary organ fields or sub-fields which control the development of the notochord along the mid-line and of the first-formed mesodermal structures which produce body cavity, excretory and muscular systems on either side of this. It also, by way of the medullary plate, induces the formation of the sub-fields concerned with the formation of the nose, the ear and the eye, anterior to and lateral to the brain region.

In this way the embryo becomes a mosaic of different fields, each a centre of dynamic energy and concerned with the development of some particular organ system, eye, ear, heart, limbs, tail and so forth. This establishment of local control in different areas of the developing embryo is spoken of as *emancipation*, indicating some measure of freedom in these various areas, although all work together for the eventual formation of the unified organism. The, as yet, unspecialized embryonic cells provide the material on which the dynamic energy of the various fields exerts its effect. It does not appear unreasonable to postulate a competition between the activity of the various fields of activity, but there is no compromise; cells lying between two such fields must come completely under the domination of one or the other; once they have done so there is no turning back, they are inevitably committed to the formation of a particular organ, eye or ear or limb as the case may be. Meantime the original unitary primary field persists especially in connexion with the direction of the primary axis of growth and of the various subsidiary organ fields.

It will be profitable at this stage to consider briefly a few examples of these secondary fields and of their mode of operation. The classic case is that of the eye which will be dealt with first. It has long been known that the vertebrate eye has a dual origin. After the primitive brain has made its appearance, there develop on either side of the fore-brain a pair of projections known as the optic vesicles, which grow outwards towards the side of the head. Gradually each assumes the form of a hollow bulb attached by a stalk. The latter gives rise to the optic nerve, the former to the eye itself. The outer wall becomes pushed in against the inner wall so forming the optic cup, the outer cells of which eventually constitute the retina. But at the same time the superficial ectodermal cells nearest to the centre of the optic cup thicken, project down into the opening of the cup and then separate from the superficial ectoderm to form the rudiment of the future lens. The completed eye, therefore, represents the intimate functional association of material from two sources.

Here is an organ rudiment admirably suited for experimental treatment and experimental embryologists have been quick to avail themselves of the opportunity. In the Amphibia the results obtained have varied according to the species studied. In the grass frog, *Rana fusca*, if the eye rudiment is removed not only does no optic vesicle appear but also no lens, although the cells which normally form this are uninjured. This indicates that the optic vesicle induces the formation of the lens and this conclusion is confirmed by the results of experiments involving the removal of the optic rudiment and its transplantation under the ectoderm of the body, when a lens appears, for instance, in the middle of the under side of the body. In the same way if the ectoderm which normally overlies the optic vesicle is removed and replaced by grafted cells from other parts of the body a lens is formed. In other words there is, in this species, nothing inherent in the ectoderm which brings about the formation of a lens ; this is induced by the specific organizing action of the cells which form the optic vesicle. On the other hand in the allied edible frog, *Rana esculenta*, or in the axolotl, the lens does appear even after the removal of the vesicle below it, while grafting of the eye rudiment into other regions of the body does not induce the appearance of a lens in those regions. Other examples could be quoted where conditions are intermediate between these two extremes, and the explanation of the differences, although the evidence is not yet complete, appears to reside in the time at which chemical, as apart from structural, differentiation of the ectodermal cells occurs in different species. Once this has occurred there is no going back ; lens formation of certain cells, skin formation by other cells is from that moment irrevocably determined.

In the same way the formation of the capsule of skeletal material which surrounds the ear does not occur if the rudiment of the ear vesicle is extirpated in the early embryo. That the capsule is formed as a result of inductive action is proved by its formation in unusual regions of the embryo into which the rudiment of the ear vesicle have been transplanted.

Although each organ field is normally an entity it may combine with another of the same type, or, on the other hand, if a particular organ rudiment is divided it may give rise to two or more similar structures instead of the usual one. A few examples will make these points clear. If an eye rudiment in a sufficiently early stage is transplanted close to another at about the same stage in development, due attention being paid to the orientation of the graft, the two organ fields will fuse with the consequent formation of a single, although rather large, eye. Such a fusion of separate fields would appear to occur in the normal development of the vertebrate heart. This organ arises in the trunk region from the mesodermal plates on either side of the mid-line ventrally. The rudiments grow together and eventually form a single tubular structure which divides up into the four primitive chambers, which early begin those rhythmical pulsations which will be continued throughout the life of the animal. It is now some three hundred years ago since Harvey spoke of the "capering bloody point" of the embryonic heart. If the early heart rudiments are transplanted a four-chambered pulsating heart may be formed in altogether abnormal parts of the body. But if the mesodermal rudiments from the two sides are prevented from growing together, by the introduction between them of foreign tissue or the cutting of a wide incision, then each will proceed to form a separate heart with, in due course, circulatory powers. Thus both in normal development and as a result of experimental grafting two like centres of developmental activity may blend with the resultant formation of a single structure.

Subdivision of organ fields has been brought about in many instances. With the heart, the two original components may be kept separate and each subdivided with the consequent formation of as many as five hearts in a single embryo. Limb rudiments may be split and twin limbs produced. It is probable that abnormalities in development which cause separation of organ rudiments are responsible for the birth of animals with additional limbs, eyes and even, on occasion, heads.

It is only possible to consider one further point which emerges from the experimental analysis of limb formation. This is controlled by a bud of mesodermal cells which contain the specific organizing field, the outer, ectodermal, cells merely conforming to the growth pattern assumed by these underlying cells. Transplantations of these mesodermal buds beneath the ectoderm elsewhere and the consequent formation there of a limb proves this. But in the case of the limb we are dealing with an organ having considerable extension in length and which demonstrates very beautifully that gradient in activity to which reference was made earlier. If the limb of a newt is amputated at the base, the wound heals and then, after a few weeks, a mass of cells accumulates under the epidermal covering. This cell mass, or blastema, grows and gradually assumes the form of a limb with all the internal equipment of bones and muscles and surrounded by skin. Into this new limb penetrate blood vessels and nerves to nourish and control it.

Now if the limb be severed in the region of the forearm and then the humerus be removed from the portion which is left, the blastema which subsequently appears is able to regenerate the lower half of the limb including the digits, but is incapable of forming a new humerus behind the cut. This surely indicates the presence of a field of activity possessing a gradient of activity from the base outwards. Regeneration from the base produces a complete new limb; regeneration from an intermediate position along the limb produces only the distal portion, even although constituents from the more proximal regions have been removed, because only a part of the organ field is concerned. There is much in the facts of regeneration amongst the simpler invertebrates which supports this theory of gradients in activity, which was originally developed by Professor C. M. Child as a result of work on regeneration which largely preceded modern developments in experimental embryology. Regeneration essentially represents embryonic development during adult life and the incorporation of its study within the sphere of experimental embryology has led to an enrichment of the content of that branch of zoology.

It is impossible to proceed further with this subject and to discuss the specialization of cells to form tissues and the later appearance of functional organs. Here both the organ field and the interaction of tissues play their part, while functional activity itself finally assumes some control over maintenance or "conservation" of fully constituted organs or tissues. But despite the heterogeneous nature of the adult structure produced by this mosaic of developmental fields, there is finally produced that unit in function and behaviour which we designate an organism.

In this brief survey of recent developments in experimental embryology an attempt has been made to indicate the range and far-reaching significance of modern investigations. Embryology, which at the beginning of the century was little more than descriptive repetition, has now become one of the most active branches of zoology. Modern work has, in some measure, reconciled the old conflict between Preformation and Epigenisis. There is an underlying preformation, although not of the type postulated by the old Preformationists, but development itself is epigenetic representing a progressive increase in complexity from egg to adult. The experimental analysis of development is one of the most hopeful approaches to that fuller interpretation of living things and living processes which is the essential aim of the science of zoology. In that way it is adding to the basic raw material of knowledge which the art and science of medicine applies to the amelioration of human suffering and the promotion of the health and happiness of mankind.

